# RAMESES publication standards: meta-narrative reviews

**DOI:** 10.1186/1741-7015-11-20

**Published:** 2013-01-29

**Authors:** Geoff Wong, Trish Greenhalgh, Gill Westhorp, Jeanette Buckingham, Ray Pawson

**Affiliations:** 1 Centre for Primary Care and Public Health, Queen Mary University of London, 58 Turner Street, London E1 2AB, UK; 2 Community Matters, P.O. Box 443, Mount Torrens, SA 5244, Australia; 3 John W. Scott Health Sciences Library, University of Alberta, Edmonton, AB T6G 2R7, Canada; 4 Department of Social Research Methodology, University of Leeds, Leeds LS2 9JT, UK

**Keywords:** meta-narrative review, meta-narrative synthesis, publication standards

## Abstract

**Background:**

Meta-narrative review is one of an emerging menu of new approaches to qualitative and mixed-method systematic review. A meta-narrative review seeks to illuminate a heterogeneous topic area by highlighting the contrasting and complementary ways in which researchers have studied the same or a similar topic. No previous publication standards exist for the reporting of meta-narrative reviews. This publication standard was developed as part of the RAMESES (Realist And MEta-narrative Evidence Syntheses: Evolving Standards) project. The project's aim is to produce preliminary publication standards for meta-narrative reviews.

**Methods:**

We (a) collated and summarized existing literature on the principles of good practice in meta-narrative reviews; (b) considered the extent to which these principles had been followed by published reviews, thereby identifying how rigor may be lost and how existing methods could be improved; (c) used a three-round online Delphi method with an interdisciplinary panel of national and international experts in evidence synthesis, meta-narrative reviews, policy and/or publishing to produce and iteratively refine a draft set of methodological steps and publication standards; (d) provided real-time support to ongoing meta-narrative reviews and the open-access RAMESES online discussion list so as to capture problems and questions as they arose; and (e) synthesized expert input, evidence review and real-time problem analysis into a definitive set of standards.

**Results:**

We identified nine published meta-narrative reviews, provided real-time support to four ongoing reviews and captured questions raised in the RAMESES discussion list. Through analysis and discussion within the project team, we summarized the published literature, and common questions and challenges into briefing materials for the Delphi panel, comprising 33 members. Within three rounds this panel had reached consensus on 20 key publication standards, with an overall response rate of 90%.

**Conclusion:**

This project used multiple sources to draw together evidence and expertise in meta-narrative reviews. For each item we have included an explanation for why it is important and guidance on how it might be reported. Meta-narrative review is a relatively new method for evidence synthesis and as experience and methodological developments occur, we anticipate that these standards will evolve to reflect further theoretical and methodological developments. We hope that these standards will act as a resource that will contribute to improving the reporting of meta-narrative reviews.

To encourage dissemination of the RAMESES publication standards, this article is co-published in the Journal of Advanced Nursing and is freely accessible on Wiley Online Library (http://www.wileyonlinelibrary.com/journal/jan).

Please see related articles http://www.biomedcentral.com/1741-7015/11/21 and http://www.biomedcentral.com/1741-7015/11/22

## Background

Academics and policymakers are increasingly interested in summarizsing the literature on complex questions that have been considered from different angles by different groups of researchers. The field of secondary research is expanding rapidly. A number of relatively new approaches are available to those seeking to undertake 'mixed method' literature reviews that combine qualitative and quantitative evidence, explore the nature and impact of complex interventions, and identify the mechanisms by which programs achieve their goals (or why they fail to do so) [1-3]. These approaches seek not only to address the questions 'what works?' and 'what is the effect size?' but to illuminate and clarify a complex topic area and highlight the strengths and limitations of different research approaches to that topic [4]. One such approach is meta-narrative review.

### What is a meta-narrative review?

Meta-narrative review is a relatively new method of systematic review, designed for topics that have been differently conceptualized and studied by different groups of researchers. For example, many different groups have, for different reasons and in different ways, studied the building of dams in India. Some have conceptualized this dam-building as engineering; others as colonialism; others as a threat (or promise) to the local eco-system; others as inspiration for literature and drama, and so on. If we were to summarize this topic area in a way that was faithful to what each different group set out to do, we would have to start by asking how each of them approached the topic, what aspect of 'dams in India' they chose to study and how. In order to understand the many approaches, we would have to consciously and reflexively step out of our own world-view, learn some new vocabulary and methods, and try to view the topic of 'dams in India' through multiple different sets of eyes. When we had begun to understand the different perspectives, we could summarize them in an over-arching narrative, highlighting what the different research teams might learn from one another's approaches.

The methodology of meta-narrative review was developed by Greenhalgh *et al*. in 2004 as a pragmatic response to challenges that emerged in a review on diffusion of service-level innovations in healthcare [5]. A methods paper was published in early 2005 [6]. The inspiration for the method was Kuhn's 1962 book *The Structure of Scientific Revolutions*, which argued that science progresses in paradigms (that is, particular ways of viewing the world, including assumptions about how the world works) and that one scientific paradigm gives way to another as scientific progress renders yesterday's assumptions and practices obsolete [7]. Newton's theories and methods, for example, became less and less able to answer the emerging questions of particle physics, leading Einstein to develop his theory of relativity. Meta-narrative review looks historically at how particular research traditions have unfolded over time and shaped the kind of questions being asked and the methods used to answer them. According to Kuhn, a research tradition is a series of linked studies, each building on what has gone before and taking place within a coherent paradigm (that is, within a particular set of assumptions and preferred methodological approaches that are shared by a group of scientists at a particular point in time).

The meta-narrative approach has some parallels to what Paterson *et al*. call the 'meta-theoretical' method [8], but is more closely aligned to 'meta-triangulation', another Kuhnian approach which we came across when researching the background for the RAMESES project [9]. The similarities and differences between meta-narrative and meta-triangulation approaches are shown in Additional file 1. In short, meta-triangulation review has a more theoretical focus and is not principally concerned with informing policy decisions. Meta-theoretical review focuses more narrowly on comparing the theoretical basis of empirical studies.

### Why are publication standards needed?

Publication standards are common (and, increasingly, expected) in health services research - see for example, CONSORT for randomized controlled trials [10], AGREE for clinical guidelines [11], PRISMA for Cochrane-style systematic reviews [12] and SQUIRE for quality improvement studies [13]. For meta-narrative reviews, publication standards are urgently needed as this method is increasingly popular and we have encountered examples of inappropriate application of the methodology in papers, theses and grant applications, which we have been asked to review. Publication standards are needed to ensure that users of reviews are provided with relevant and necessary information to enable them to assess the quality and rigor of a review.

In our experience, there is considerable confusion among researchers, journal editors, peer reviewers and funders about what counts as a high quality meta-narrative review and what, conversely, counts as a flawed review. Even though experts still differ on detailed conceptual methodological issues, the increasing popularity of this method prompted a study to develop baseline standards from which, we anticipate, further developments in theory and methodology of this approach will occur.

## Aim

The aim of this paper is to produce preliminary publication standards for meta-narrative reviews.

## Method

The methods we used to develop these reporting standards have already been published [14]. In brief, we purposively recruited an international group of experts to our online Delphi panel. Aiming to achieve maximum variety in the relevant sectors, disciplines and expert perspectives represented, we sought panel members working in meta-narrative reviews, evidence synthesis, publication, reviewer training and health policy. Prior to the start of our Delphi panel, with input from an expert informaticist (JB), we collated and summarized existing literature on the principles of good practice in meta-narrative reviews, created a database of such published reviews, and built relationships with teams who were undertaking ongoing reviews. Through discussion within the project team, we considered the extent to which the principles had been followed by published and in-progress reviews, thereby identifying how rigor may be lost and how existing methods could be improved.

Our analysis of existing meta-narrative reviews formed the basis of the briefing materials for the first round of the Delphi panel. In addition, we drew on our collective experience in training and supporting meta-narrative review teams and an email discussion list on realist and meta-narrative methodology [15] to further inform the contents of our briefing document. Both the research team and panel members contributed draft items for the publication standards, and these were refined using the online Delphi process as previously described [14]. We ran the Delphi panels between September 2011 and March 2012.

### Description of panel and items

In all, we recruited 33 individuals from 25 organizations in six countries. These comprised researchers in public or population health researchers (5); evidence synthesis (5); health services research (8); international development (2); education (2); and also research methodologists (6), publishing (1), nursing (2) and policy and decision making (2). In round 1, 22 panel members provided suggestions of items that should be included in the publication standards. In rounds 2 and 3, our panel members were asked to rate each potential item for relevance and clarity. The response rates across all items for round 2 and 3 were 93% and 87%, respectively. Consensus was reached within three rounds on both the content and wording of 20 items within the publication standards. Table 1 provides an overview of these items.

**Table 1:** List of items to be included when reporting a meta-narrative review

TITLE		
**1**		**In the title, identify the document as a meta-narrative review or synthesis**
**ABSTRACT**		
**2**		**While acknowledging publication requirements and house style, abstracts should ideally contain brief details of: the study's background, review question or objectives; search strategy; methods of selection, appraisal, analysis and synthesis of sources; main results; and implications for practice**.
**INTRODUCTION**		
**3**	Rationale for review	**Explain why the review is needed and what it is likely to contribute to existing understanding of the topic area**.
**4**	Objectives and focus of review	**State the objective(s) of the review and/or the review question(s). Define and provide a rationale for the focus of the review**.
**METHODS**		
**5**	Changes in the review process	**Any changes made to the review process that was initially planned should be briefly described and justified**.
**6**	Rationale for using meta-narrative review	**Explain why meta-narrative review was considered the most appropriate method to use**.
**7**	Evidence of adherence to guiding principles of meta-narrative review	**Where appropriate show how each of the six guiding principles (pragmatism, pluralism, historicity, contestation, reflexivity and peer review) have been followed**.
**8**	Scoping the literature	**Describe and justify the initial process of exploratory scoping of literature**.
**9**	Searching processes	**While considering specific requirements of the journal or other publication outlet, state and provide a rationale for how the iterative searching was done. Provide details on all the sources accessed for information in the review. Where searching in electronic databases has taken place, the details should include (for example) name of database, search terms, dates of coverage and date last searched. If individuals familiar with the relevant literature and/or topic area were contacted, indicate how they were identified and selected**.
**10**	Selection and appraisal of documents	**Explain how judgements were made about including and excluding data from documents, and justify these**.
**11**	Data extraction	**Describe and explain which data or information were extracted from the included documents and justify this selection**.
**12**	Analysis and synthesis processes	**Describe the analysis and synthesis processes in detail. This section should include information on the constructs analysed and describe the analytic process**.
**RESULTS**		
**13**	Document flow diagram	**Provide details on the number of documents assessed for eligibility and included in the review with reasons for exclusion at each stage as well as an indication of their source of origin (for example, from searching databases, reference lists and so on). You may consider using the example templates (which are likely to need modification to suit the data) that are provided**.
**14**	Document characteristics	**Provide information on the characteristics of the documents included in the review**.
**15**	Main findings	**Present the key findings with a specific focus on theory building and testing**.
**DISCUSSION**		
**16**	Summary of findings	**Summarise the main findings, taking into account the review's objective(s), research question(s), focus and intended audience(s)**.
**17**	Strengths, limitations and future research directions	**Discuss both the strengths of the review and its limitations. These should include (but need not be restricted to) (a) consideration of all the steps in the review process and (b) comment on the overall strength of evidence supporting the explanatory insights which emerged**. **The limitations identified may point to areas where further work is needed**.
**18**	Comparison with existing literature	**Where applicable, compare and contrast the review's findings with the existing literature (for example, other reviews) on the same topic**.
**19**	Conclusion and Recommendations	**List the main implications of the findings and place these in the context of other relevant literature. If appropriate, offer recommendations for policy and practice**.

**20**	Funding	**Provide details of funding source (if any) for the review, the role played by the funder (if any) and any conflicts of interests of the reviewers**.

### Scope of the publication standards

These publication standards are intended to help researchers, authors, journal editors and policy and decision makers to know and understand what should be reported in the write-up of a meta-narrative review. They are not intended to provide detailed guidance on how to conduct such a review; for this we direct interested readers to other publications [5,6]. This publication standard applies only to meta-narrative reviews. A list of publication guidelines for other review methods can be found on the EQUATOR Network's website [16], but at present none of these relate specifically to meta-narrative reviews. As part of the RAMESES project we are also developing quality standards and training materials for meta-narrative review, which will be submitted as a separate publication. Publication standards for realist syntheses (also covered in the RAMESES project) have been addressed in a separate article.

### How to use these publication standards

The layout of this document has drawn on previous methodological publications and in particular on the 'Explanations and Elaborations' document of the PRISMA statement [12]. Each item is followed by an example drawn from published reviews and a rationale for its inclusion. The purpose of the example text is to illustrate how an item might be reported in a write-up. However, potentially relevant contextual information may have been omitted, so it may be necessary to consult the original paper from which the example text was drawn. The standards set out what might be expected for each item, but authors will still need to exercise judgement about how much information to include. The purpose of the detail reported should be to ensure that the description and explanation provided is coherent and plausible, both against the guidance set out within an item and for the overall purpose of the meta-narrative review.

While this publication standard is modeled on the PRISMA statement, the items within are not identical. This publication standard, developed to apply only to meta-narrative reviews, has some overlap with the PRISMA statement. Items 1 to 3, 16, 17 and 20 in this statement broadly match the purpose of items 1 to 3, 24, 25 and 27 in the PRISMA statement. For items 4 to 15, while there is some overlap in purpose with some PRISMA statement items, different or additional reporting is needed due to the nature of meta-narrative reviews. Other items (5, 12, 13, 15, 16, 19 and 23) in the PRIMSA statement have no equivalent in the RAMESES publication standards for realist reviews.

The order in which items are reported may vary. Meta-narrative reviews are not 'linear' reviews. Some of the processes that are listed may legitimately take place in parallel or have to be revisited at a later date as a review progresses. As a general rule, if a recommended item is excluded from the write-up of a meta-narrative review, a justification should be provided.

## The RAMESES publication standards for meta-narrative reviews

### Item 1: Title

In the title, identify the document as a meta-narrative review or synthesis.

### Example

"Tensions and Paradoxes in Electronic Patient Record Research: A Systematic Literature Review Using the Meta-narrative Method." [17]

### Explanation

Some meta-narrative reviews are not flagged as such in the title and/or are inconsistently indexed and, hence, are difficult to locate in searches. Most authors currently use the term 'meta-narrative review'. No consensus was reached by our Delphi panel on whether 'review' or 'synthesis' should be the preferred term, and there seemed no good reason to impose one or the other term.

#### Item 2: Abstract

While acknowledging that requirements and house style may differ between journals, abstracts should ideally contain brief details of the study's background, review question or objectives; search strategy; methods of selection, appraisal, analysis and synthesis of sources; main results; and implications for practice.

### Example

"Background: The therapeutic relationship is complex. Any attempt to capture its quality in a score or metric must involve an element of reductionism. But policymakers increasingly ignore the unmeasured.

Aim: To review the different concepts, theoretical models and empirical approaches which researchers have used to capture the relationship between practitioner and patient in terms of scales, categories and other objective metrics.

Method: Drawing on the principles of meta-narrative systematic review (but without seeking an exhaustive inventory of every paper ever published), we considered different research traditions in terms of their respective philosophical assumptions, methodological strengths and limitations and empirical findings. We applied published quality criteria from each tradition to papers within that tradition.

Results: Three main research approaches were oriented to producing objective data about the therapeutic relationship. These appeared to have emerged in different research traditions: patient satisfaction surveys (health services research), rate-your-relationship surveys (social psychology) and interaction analysis (cognitive psychology). Each emphasised a different dimension and produced a different perspective on quality.

Conclusions: Objective metrics, when well designed, offer important insights into the therapeutic relationship, but its elusive essence remains imperfectly captured by the best of them." [18]

### Explanation

Apart from the title, an abstract is the only source of information accessible to searchers unless the full paper is obtained. The information in it must allow reviewers and/or users to decide if the review is relevant to their needs.

## Introduction section

The following items should be reported in the introduction section.

### Item 3: Rationale for review

Explain why the review is needed and what it is likely to contribute to existing understanding of the topic area.

### Example

"A number of researchers have documented a tremendous gap between knowledge and policy action to tackle social gradients in health (References x8). Yet, the roles and capacities of urban municipalities to address population health inequities, as perceived by both researchers and urban municipal policy-makers themselves, have been particularly neglected areas of study. While the Healthy Cities movement has been active in prescribing avenues for municipal activity (primarily in non-academic/grey literature (References x4)), it remains to be empirically demonstrated how other health inequities literatures have implicated municipalities, the precise nature of these implications, and the manner in which these implications are taken up by relevant municipal actors and institutions." [19]

### Explanation

As with all research, a background section explaining what is already known and what the researchers considered the 'knowledge gaps' to be is a helpful orientation.

#### Item 4: Objectives and focus of review

State the objective(s) of the review and/or the review question(s). Define and provide a rationale for the focus of the review.

### Example

....“our review is focused on the *collective level of analysis *in order to understand *deliberate interventions *aimed at influencing behaviors or opinions though the *communication of information*." [20]

### Explanation

A meta-narrative review asks some or all of the following questions:

(1) Which research (or epistemic) traditions have considered this broad topic area?; (2) How has each tradition conceptualized the topic (for example, including assumptions about the nature of reality, preferred study designs and ways of knowing)?; (3) What theoretical approaches and methods did they use?; (4) What are the main empirical findings?; and (5) What insights can be drawn by combining and comparing findings from different traditions?'

Because a meta-narrative review may generate a large number of avenues that might be explored and explained, and because resources and timescale are invariably finite, the expectation is that the review must be 'contained' by progressively focusing both its breadth (how wide an area?) and depth (how much detail?). This important process may involve discussion and negotiation with (for example) content experts, funders and/or users. It is typical and legitimate for the review's objectives, question and/or the breadth and depth of the review to evolve as the review progresses. How and why it evolved is usually worth reporting.

### Methods section

The following items should be reported in the methods section.

#### Item 5: Changes in the review process

Any changes made to the review that were initially planned should be briefly described and justified.

### Example

"But as the review unfolded, two things became clear: first, in many areas, the evidence meeting all these criteria was sparse, and second, we could gain critical insights from beyond the parameters we had set. We therefore extended our criteria to a wider range of literature. In particular, we added both overview articles and "landmark" empirical studies from outside the health sector if they had important methodological or theoretical lessons for our research question." [5]

### Explanation

A meta-narrative review can (and, in general, should) evolve over the course of the review. For example, changes to the research question or its scope are likely to have an impact on many of the review's subsequent processes. However, this does not mean the review can meander uncontained. An accessible summary of what was originally planned (for example, as described in an initial protocol) and how and why this differed from what was done should be provided as this may assist interpretation.

#### Item 6: Rationale for using the meta-narrative approach

Explain why meta-narrative review was considered the most appropriate method to use.

### Example

"We used an adaptation of meta-narrative review, based on Kuhn's notion of the scientific paradigm (a coherent body of work that shares a common set of concepts, theories, methods and instruments).(references x2) This qualitative approach seeks to tease out the over-arching storylines of different research traditions by asking four key questions: how is the topic conceptualised in each separate tradition?; what are the key theory(ies)?; what are the preferred study designs and ways of knowing? and what are the main empirical findings? Meta-narrative review is pluralistic rather than normative (ie, it asks not 'what is the best approach to researching this topic?' but 'what can we learn from the range of different approaches?'). It is particularly suited to exploring tensions and paradoxes between different research traditions and making sense of 'conflicting' findings." [18]

### Explanation

Meta-narrative review, (which is rooted in a constructivist philosophy of science), is inspired by the work of Thomas Kuhn, who observed that science progresses in paradigms (see definition below). Meta-narrative reviews often look historically at how particular research traditions or epistemic traditions have unfolded over time and shaped the 'normal science' of a topic area.

### Some definitions

• A **paradigm **is a particular way of viewing the world, including assumptions about how the world works, what are the important questions in a particular topic area, and what study designs and methods are best for adding to the knowledge base.

• A **research tradition **comprises studies building on what has gone before, each building on what has gone before, usually situated within a coherent paradigm, though an interdisciplinary tradition may bridge more than one paradigm.

• An **epistemic tradition **is the unfolding of the underpinning set of philosophical assumptions which drive the development of theory and method; scholarship may progress via debate around these assumptions even in the absence of new empirical studies.

• **Normal science **is a paradigm along with the practices and empirical approaches which are taken for granted by scientists within a particular tradition.

Meta-narrative review is, therefore, best suited to studying topic areas that have been differently conceptualized and studied by different groups. The review seeks first to identify and understand as many as possible of the potentially important different research traditions which have a bearing on the topic, and then to synthesize them by means of an over-arching narrative. The goal of meta-narrative review is sensemaking of a complex (and perhaps contested) topic area.

#### Item 7: Evidence of adherence to guiding principles of meta-narrative review

Where appropriate, show how each of the six guiding principles (pragmatism, pluralism, historicity, contestation, reflexivity and peer review) have been followed.

### Example

"We identified 13 research areas that had, largely independently of one another, provided evidence relevant to the diffusion of innovations in health service organizations (Table 1). Four of these traditions can be classified as "early diffusion research”:

.... One important weakness of the literature on structural determinants of innovativeness is the assumption that they can be treated as variables whose impact can be isolated and independently quantified. For example, the empirical studies of organizational size implicitly assume that there is a "size effect" that is worth measuring and that is to some extent generalizable. An alternative theoretical approach (Reference x1), supported by a number of recent detailed qualitative studies (References x2), is that the determinants of organizational innovativeness interact in a complex, un-predictable, and nongeneralizable way with one another." [5]

### Explanation

Currently meta-narrative review is based on six guiding principles [6]:

• Principle of pragmatism: what to include is not self-evident. The reviewer must be guided by what will be most useful to the intended audience(s), for example, what is likely to promote sense making;

• Principle of pluralism: the topic should be illuminated from multiple angles and perspectives, using the established quality criteria appropriate to each. For example, reviewers should avoid beginning with a single 'preferred' perspective or methodological hierarchy and proceed to judge work in other traditions using these external benchmarks. Research that lacks rigor must be rejected, but the grounds for rejection should be intrinsic to the relevant tradition, not imposed on it;

• Principle of historicity: research traditions are often best described as they unfolded over time, highlighting significant individual scientists, events and discoveries which shaped the tradition;

• Principle of contestation: 'conflicting data' from different research traditions should be examined to generate higher-order insights (for example, about how different research teams framed the issue differently or made different assumptions about the nature of reality);

• Principle of reflexivity: throughout the review, reviewers must continually reflect, individually and as a team, on the emerging findings;

• Principle of peer review: emerging findings should ideally be presented to an external audience and their feedback used to guide further reflection and analysis.

The published literature on meta-narrative review indicates that some review teams have deliberately adapted the method as first described by Greenhalgh *et al*. [6]. While evolution and/or adaptation of the method is to be welcomed in principle, the description and rationale for any adaptations made should be provided to allow readers to judge their appropriateness.

#### Item 8: Scoping the literature

Describe and justify the initial process of exploratory scoping of the literature.

### Example

....“we undertook an initial 'territory mapping' exercise. We each explored a different area of possibly relevant research using informal and unstructured methods. We asked colleagues, sent emails to academic lists, browsed libraries and the Internet, and built on our own prior knowledge. One of us began, for example, with the literature on evidence-based medicine (EBM) and guideline implementation (Reference x1), which led serendipitously to another literature on health promotion campaigns (Reference x1) (the spread of 'innovative messages' about healthy lifestyles). One of us was directed by a colleague towards work on technology transfer to developing countries (Reference x1), and discovered a huge 'grey literature' in the databases of international development agencies. Another had previously completed a PhD that involved exploring social network theory in relation to the spread of medical technologies (Reference x1). By exploring all these (and more) avenues, we gained a feel of the overall literature." [6]

### Explanation

One of the main challenges in meta-narrative review is to identify a sufficiently broad range of sources so as to be able to build as comprehensive a map as possible of research undertaken on the topic. This scoping step is used to identify in broad terms the different research traditions, situated in different literatures, which have addressed the topic of interest. Initial attempts to make sense of a topic area may involve not just informal 'browsing' of the literature but also consulting with experts and stakeholders.

#### Item 9: Searching process

While considering specific requirements of the journal or other publication outlet, state and provide a rationale for how the iterative searching was done. Provide details on all the sources accessed for information in the review. For example, where electronic databases have been searched, details should include, for example, names of databases, search terms, dates of coverage, and dates last searched. If individuals familiar with the relevant literature and/or topic area were contacted, indicate how they were identified and selected.

### Example

"Inspired in a large part by the work of Greenhalgh and colleagues (Reference x2), we relied instead on a non-keyword-based reviewing process that we dubbed *double-sided systematic snowball*.

Our goal was to identify documents that made a core contribution, either conceptually or empirically, to the understanding of the phenomenon. Our starting point was to identify, through team consensus, some seminal papers (*n *= 33) that were considered to have shaped the evolution of the field. We started by identifying a heuristic list of seven "traditions”:....

Each tradition was exemplified by one or more publications. The definition of "traditions" and the identification of specific publications were interdependent processes conducted on a consensus basis. At the end of the process, we had produced a list of thirty-three "seminal" sources (see the appendix). ....

We then used the ISI Web of Science Citation Index to identify all documents (*n *= 4,201) that cited those seminal papers. The snowball process here was prospective, since it exclusively targeted documents published after the selected seminal paper. We then triaged the results using the titles and (if present) the abstracts, using a decision grid based on the definition of the phenomenon under review, as discussed in the previous section. ....

Next we used the bibliographies of those 102 documents as a basis for retrospective systematic snowball sampling. We entered each document's complete bibliography in a database (*n *= 5,622) and used algorithms to identify all articles cited five times or more and all books cited seven times or more. ....

Among the articles, we excluded fourteen based on relevance criteria and twelve that were already among the 102 identified in the first step. Finally, we included forty-nine other documents either through deliberate selection during the first step of analysis because of their empirical or conceptual contribution, or through nonsystematic sampling of the field." [20]

### Explanation

Searching should be guided by the objectives and focus of the review, and revised iteratively in the light of emerging data. By definition, a meta-narrative review seeks to identify and combine different research traditions, hence different search strategies will need to be developed as appropriate to the different literatures. This stage is likely to involve searching for different kinds of data in different ways.

Search methods using forward and backward citation tracking may be particularly valuable in finding key documents. In particular, potential seminal sources (conceptual, theoretical or empirical studies, which have defined the tradition and inspired later work) may be identified from judicious searching of the reference lists of later studies. Once identified, seminal sources should be citation-tracked to identify further sources which drew on these.

Meta-narrative reviews do not approach the literature with a pre-defined 'preferred' study design. Rather, any preferred study design(s) should be identified from quality standards developed within a particular research tradition. 'Methodological filters' (for example, to identify randomized controlled trials) should be used only when these have been designated as a quality feature by the scientists within that tradition.

Searching is necessarily iterative, since the reviewer must move between the seminal source(s) and papers which subsequently cited that source, so as to build a picture of how research unfolded in each tradition. The process used for any such additional searches should be clearly documented. A single pre-defined search is unlikely to be sufficient and may suggest insufficient reflection on emerging findings.

Sufficient detail should be given to enable the reader to judge whether searching was likely to have located sources needed for elucidating all the key research traditions.

#### Item 10: Selection and appraisal of documents

Explain how judgements were made about including and excluding data from documents, and justify these.

### Example

"Abstracts had to mention, in some capacity, differences in health outcomes or well-being, and/or the SDOH [social determinants of health]. Abstracts that discussed policy implications were also of distinct interest for review, but this was not an explicit inclusion criterion. Abstracts that described health differences in a strictly clinical scope were excluded, as were abstracts that referred to inequalities or disparities in a different context (e.g., measurement disparities). Highly technical pieces that discussed new clinical technologies, or issues related to healthcare systems and/or delivery, were excluded. Abstracts were also excluded if they contained the words "National Population Health Survey" or "Ottawa Charter for Health Promotion", but lacked any other information relevant to the review." [19]

### Explanation

Meta-narrative review is not a technical process - that is, following a set protocol will not guarantee that a review will be robust. Rather, it is a process of sense-making of the literature, selecting and combining data from primary sources to produce an account of how a research tradition unfolded and why, and then (in a second phase) comparing and contrasting findings from these different traditions to build a rich picture of the topic area from multiple angles. This process requires a series of judgements about the unfolding of research in particular traditions, and about the relevance and robustness of particular data within that tradition.

Meta-narrative review takes its quality criteria from the traditions included in the review, and in particular from seminal papers which have been accepted by others within that tradition as authoritative. A meta-narrative review might, for example, include a meta-narrative from clinical epidemiology in which randomized controlled trials and meta-analyses of these are greatly valued; it might also include a meta-narrative from critical sociology in which theory-driven qualitative studies are greatly valued. Studies in these separate traditions should be appraised using the quality criteria that a competent peer-reviewer in that tradition would choose to use.

The description of the selection and appraisal process should be sufficiently detailed to enable a reader to judge how likely it is that researchers inadvertently excluded data that may have significantly altered the findings of the review.

#### Item 11: Data extraction

Describe and explain which data or information were extracted from the included documents and justify this selection.

### Example

"Bibliographic characteristics of interest were body of literature .... from which the abstract was retrieved; journal name; publication year; geographical region of focus (or origin); type of study described in the abstract; and population investigated by the study or target audience. Abstract contents were captured using two variables: article themes and SDOH [social determinants of health] profile. Article theme codes were developed through an inductive process of immersion with the article abstracts and saturation of article themes; codes were based not on any one particular keyword or phrase in the abstracts, but on the content area as conveyed by the abstract as a whole." [19]

### Explanation

The type of data collected in meta-narrative review can be very diverse. The analysis and synthesis phases are influenced by the amount and type of data extracted. Reporting on what was extracted and why can add to the transparency of the review process.

In a meta-narrative review the data elements extracted would go to constructing a story of how research on a topic unfolded over time in a particular tradition. This may include (where relevant), for example:

• upstream (antecedent) traditions from which these emerged; background philosophical assumptions;

• research questions and how they were framed; conceptual and theoretical issues;

• preferred methodologies, study designs and quality criteria;

• key actors (for example, leading scientists or commentators) and events (for example, conferences) in the unfolding of the tradition;

• landmark empirical or theoretical studies;

• significant findings and how these shaped subsequent work; and

key debates and areas of dispute within the tradition, including links with or breaches from other traditions.

Meta-narrative review is used for a wide range of research questions, so it is impossible to be prescriptive about which data should be extracted. However, the link between the research question and the type of data extracted should be clear.

#### Item 12: Analysis and synthesis processes

Describe the analysis and synthesis processes in detail. This section should include information on the process by which the account of each meta-narrative (that is, the story of each unfolding research tradition) was built up and how the separate meta-narratives were compared and contrasted. Document and justify any changes in this process as the study unfolded.

### Example

"We mapped the meta-narratives (i.e., we traced the historical development of concepts, theory, and methods in each research tradition) by identifying the seminal theoretical and overview papers and books and analyzing the conceptual and theoretical models proposed by recognized experts in each field. ....

Because different researchers in different traditions generally conceptualized their topic differently; used different language and metaphors for diffusion, dissemination, and implementation; asked different questions; privileged different methods; and used different criteria to judge "quality" and "success," we used narrative, rather than statistical, synthesis techniques. ....

We highlighted the similarities and differences of the findings from different research traditions and considered the reasons for the differences. In this way, the heterogeneity of approaches and "contradictions" in findings could be turned into data and analyzed systematically." [5]

### Explanation

If exploration of a range of research traditions on the topic is not deemed to be appropriate, the work is probably not a meta-narrative review.

A meta-narrative review should include two specific stages, though these will usually overlap as they will necessarily influence one another iteratively.

In the analysis stage, reviewers should seek to identify and map out specific meta-narratives (that is, unfolding stories of research traditions over time), focusing in particular on the concepts, theories, methods and instruments which have characterized the tradition, major findings in that tradition and foci of dissent and disagreement.

The process of building this unfolding storyline is essentially interpretive and, hence, follows the principles of interpretivist analysis, including immersion in the data by repeated reading and/or analysis of quantitative data; reflexivity and discussion among researchers; consideration of how each new data item fits with an emerging picture of the whole; and checking where appropriate that the account is considered valid by experts within the designated research tradition. Both quantitative and qualitative traditions and data may need to be incorporated in the storyline. Explanation and justification for any analytic methods used to combine and summarize data within a particular tradition should be provided.

The synthesis stage involves comparing and contrasting the meta-narratives so as to identify and compare how the different groups have conceptualized the topic (including differences in philosophical position), how they have theorized it, and the methodological approaches and study designs used. Differences in findings between meta-narratives are higher-order data and should be analyzed interpretively to produce further insights (for example, about differences in underlying assumptions or methodological approaches between different research traditions).

Synthesis across traditions may occur at a high level of abstraction (that is, at the level of concepts and theories) and may involve one or more of the following:

• paradigm bridging (seeking commonalities in underlying conceptual and theoretical assumptions),

• paradigm bracketing (highlighting differences in these assumptions),

• interplay (exploring tensions);

• meta-theorizing (exploring patterns that span conflicting understandings)

Synthesis may also occur at a more concrete level and summarize empirical findings, using techniques including statistical aggregation, qualitative aggregation and narrative summary.

A description should be provided of how the all the individuals involved in the review have been involved in the analysis and synthesis processes, and input (if any) from external advisors/peer reviewers from included traditions.

## Results section

The following items should be reported in the Results section.

### Item 13: Document flow diagram

**Figure 1:**
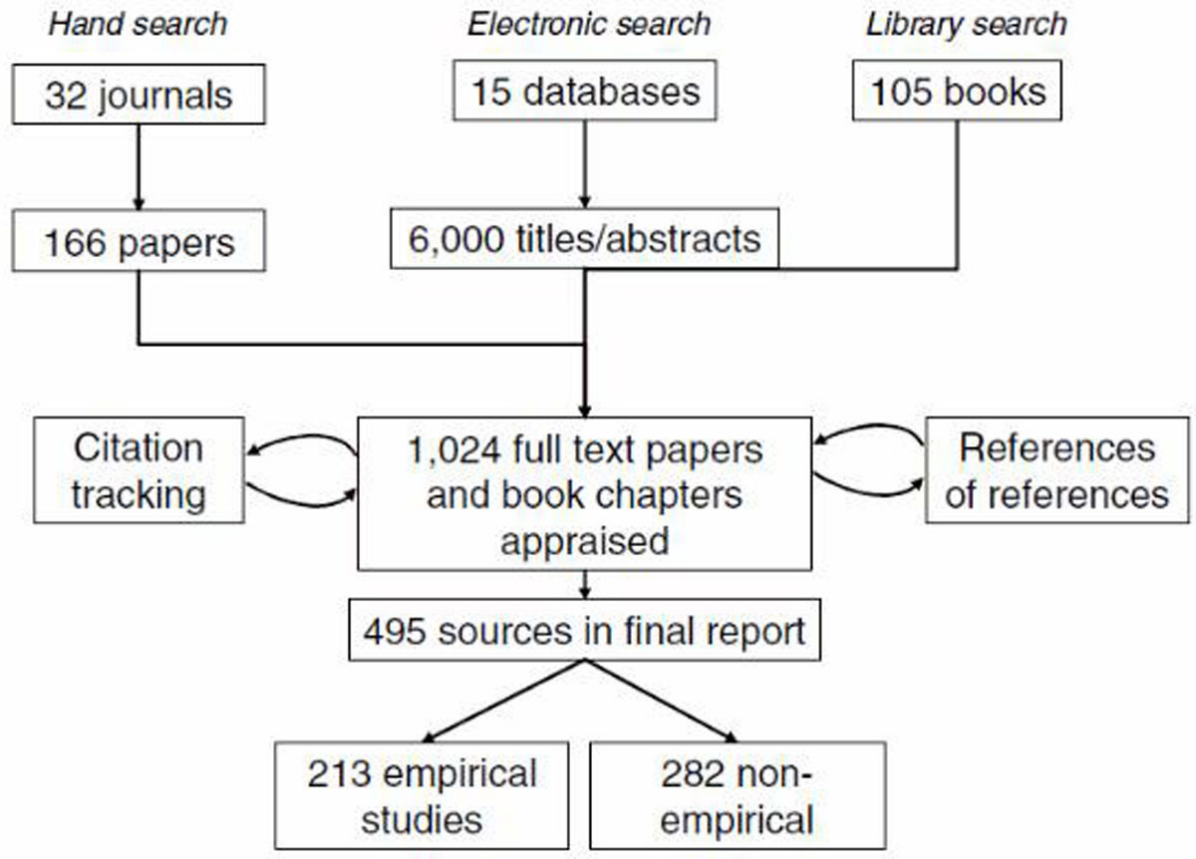
**Flow chart of search from Greenhalgh *et al***. [5].

Provide details on the number of documents assessed for eligibility and included in the review with reasons for exclusion at each stage as well as an indication of their source of origin (for example, from searching databases, reference lists and so on). You may consider using the example provided (which is likely to need modification to suit the data) in Figure 1.

### Example

"The breakdown of sources that contributed to the final report is shown in Figure 1." [5]

### Explanation

A flow diagram provides an accessible summary of the sequence of steps and gives an indication of the volume of data included and excluded at each step.

#### Item 14: Document characteristics

Provide information on the characteristics of the documents included in the review.

### Example

"The 94 primary studies (described in 129 papers) outside the health informatics literature were philosophically pluralist, with 14% positivist, 19% interpretivist, 22% critical and 55% recursive. As Table 3 shows, they also were methodologically diverse, most with different types of case studies." [17]

### Explanation

A clear summary of the characteristics of included sources can add to the transparency of the review and some characteristics may help readers judge the coherence and plausibility of inferences. Examples of possibly relevant characteristics of documents that may be worth reporting include, where applicable: full citation, country of origin, study design, summary of key main findings, use made of documents in the review and relationship of documents to each other (for example, there may be more than one document reporting on an intervention). While considering the specific requirements of any particular publication, reviewers may wish to tabulate key characteristics.

#### Item 15: Main findings

Present the key findings with a specific focus on the key meta-narratives that have a bearing on the topic area, and the commonalities and differences between them.

### Example

"Exploratory searches suggested that approaches could be divided into two broad schools ('objective' and 'subjective'). In reality there is much overlap between them for example, many 'objective' numerical scales are designed to capture and quantify respondents' subjective perceptions. The objective school defines research rigour in positivistic terms (accuracy, precision, reproducibility, inter-rater reliability and distancing from the data) while the subjective school defines rigour in interpretivist terms (strength of underpinning theory, coherence of concepts and explanations, reflexivity and immersion in the data).

The objective school .... is oriented to producing verifiable and reproducible facts (such as scores, estimates of frequencies or lists of commonly occurring themes). ....

The subjective school, oriented to generating interpretations rather than facts, includes psychodynamic analysis (e.g. Balint method), narrative analysis, critical consultation analysis and socio-technical analysis." [18]

### Explanation

The defining feature of a meta-narrative review is illumination of a complex topic area from multiple angles. In general, this will be achieved by first presenting each meta-narrative as a coherent individual account which conveys the underpinning 'normal science' of the relevant research tradition (concepts, theories, preferred methods) and the key empirical findings in that tradition. Findings and inferences from the synthesis across the different meta-narratives may then be presented as an over-arching narrative which retains the integrity of the separate research traditions but draws out what might be learned from the commonalities and differences between them.

The outputs of paradigm bridging, paradigm bracketing, interplay and meta-theorizing should be presented as appropriate to summarize the conceptual and theoretical basis of the meta-narratives. The outputs of statistical aggregation, qualitative aggregation and narrative summary of disaggregated data should be presented as appropriate to summarize the empirical findings. In each case, data from the primary documents should be presented and sourced to illustrate how inferences have been made and justify these. The more detail that is given, the more readers will be able to judge the validity of the inferences.

## Discussion section

The following Items should be reported in the discussion section.

### Item 16: Summary of findings

Summarize the main findings, taking into account the review's objective(s), research question(s), focus and intended audience(s).

### Example

"The UK NPfIT [National Programme for Information Technology] appeared to be built on six assumptions, that the EPR [Electronic Patient Record] (1) is primarily a container for information about the patient; (2) can be integrated seamlessly and unproblematically into clinical work; (3) will increase the effectiveness and efficiency of clinical work; (4) will drive changes in how staff interact with the patient and one another; (5) should replace most, if not all, forms of paper record, which are old-fashioned and limited; and (6) the more comprehensive and widely distributed it is, the more value it will add.

.... Much of the literature covered in this review suggests, conversely, that (1) the EPR may be alternatively conceptualized as an "itinerary," "organizer," or "actor”; (2) seamless integration of different EPR sys-tems is unlikely because human work will always be needed to bridge the model-reality gap and recontextualize knowledge for different uses; (3) while secondary work (audit, research, billing) may be made more efficient by the EPR, primary clinical work is often made less efficient; (4) the EPR may support, but will not drive, changes in the social order of the workplace; (5) paper will not necessarily disappear, as it offers a unique level of ecological flexibility (although workable paperless systems have been developed in one or two centers); and (6) smaller, more local EPR systems may often (though perhaps not always) be more efficient and effective than larger ones." [17]

### Explanation

In order to place the findings in the context of the wider literature and any specific policy need, it is necessary to summarize briefly what has been found. This section should be succinct and balanced, highlighting the key meta-narratives that emerged from the analysis and the key points of commonality and contestation between them. This should be done with careful attention to the needs of the main users of the review.

#### Item 17: Strengths, limitations and future research directions

Discuss both the strengths of the review and its limitations. These should include (but need not be restricted to) (a) consideration of all the steps in the review process and (b) comment on the overall strength of evidence supporting the explanatory insights that emerged.

The limitations identified may point to areas where further work is needed.

### Example

"The most important limitation of our study is in attempting to make generalizations about the applicability of potential municipal government interventions across diverse governmental forms and functions, and geographical jurisdictions. ....

Another limitation of this study was in restricting our analysis to the four bodies of literature chosen. As discussed, our decision not to include the policy sciences and social epidemiology, for instance, may have led our findings to under-represent dimensions of the health inequities knowledge base that focus on broader social welfare policies or more technically-oriented epidemiological studies documenting the scope of health inequities at the local level." [19]

### Explanation

Meta-narrative reviews may be constrained by time and resources, by the skill mix and collective experience of the research team, by the scope of the review's questions or objectives and/or by anticipated or unanticipated challenges in the data. These should be made explicit so that readers can interpret the findings in light of them. A common challenge in meta-narrative reviews is that in order to focus the review, some material is omitted at each successive stage. Some aspects of the topic area, therefore, end up being reviewed in detail and rich explanatory insights produced for these. Other aspects are neglected (relatively or absolutely). It is thus inevitable that in generating illumination, the review will also cast shadows. These should be highlighted in the discussion so as to indicate areas where other reviews might focus.

Strengths and/or limitations associated with any modifications made to the review process should also be reported and justified.

#### Item 18: Comparison with existing literature

Where applicable, compare and contrast the review's findings with the existing literature (for example, other reviews) on the same topic.

### Example

"Our review affirmed many well-described themes in the literature, such as the useful list of innovation attributes that predict (but do not guarantee) successful adoption; the importance of social influence and the networks through which it operates; the complex and contingent nature of the adoption process; the characteristics (both "hard" and "soft") of organizations that encourage and inhibit innovation; and the messy, stop-start, and difficult-to-research process of assimilation and routinization. We also exposed some demons in this literature, such as the lack of empirical evidence for the widely cited "adopter traits"; the focus on innovations that arise centrally and are disseminated through official channels at the expense of those that arise peripherally and spread informally; the limited generalizability of the empirical work on product-based innovation in companies to process innovation in service organizations; and the near absence of studies focusing primarily on the sustainability of complex service innovations." [5]

### Explanation

A meta-narrative review will typically cover a broad and diverse literature. In particular, it is likely to have uncovered findings from outside the healthcare literature (for example, sociology, cognitive or social psychology, economics, education) that may supplement and extend (and in some cases challenge) the findings of previous, more narrowly focused, systematic reviews on the topic. In general, meta-narrative reviews should make explicit where and how the review extends the knowledge base.

#### Item 19: Conclusion and recommendations

List the main implications of the findings and place these in the context of other relevant literature. If appropriate, offer recommendations for policy and practice.

### Example

"Overall, the health inequities knowledge base offered insufficient guidance to municipal governments in developing healthy public policy at the local level. Health was conceptualized in primarily 'behavioural' and 'biomedical' terms, providing little incentive for municipalities to consider, and act on, the full range of the SDOHs [social determinants of health]. If researchers, who have at their disposal voluminous evidence on the social determinants of health inequities, overwhelmingly defer to healthy lifestyles and healthcare services as the levers for improving health, then how can busy, and often uninformed, policy-makers be expected to conceptualize health any differently? The minimal attention paid to municipal governments in the health inequities knowledge base urges critical reflection on the subject areas and types of health research that funding agencies privilege, and highlights the need for increased funding and translation of interdisciplinary health inequities research that is relevant to policy-makers, especially at the municipal level where human resources devoted to exchange with research communities are in short supply." [19]

### Explanation

A clear line of reasoning is needed to link the findings (results section) with the implications (discussion and/or conclusion). If the review is small and preliminary, or if the coherence and plausibility of evidence behind the inferences is weak or moderate, statements about implications for practice and policy should be appropriately guarded.

#### Item 20: Funding

Provide details of funding source (if any) for the review, the role played by the funder (if any) and any conflicts of interests of the reviewers.

### Example

"This review had multiple funding streams, including the National Institute for Health Research Service Delivery and Organisation Programme (project numbers 08/1602/131 and 08/TA252), the Medical Research Council (project number 07/133), and the UK Department of Health via the Connecting for Health Evaluation Programme (project numbers CFHEP 002 and 007)." [17]

### Explanation

The source of funding for a review and/or personal conflicts of interests may influence the research question, methods, data analysis and conclusions. No review is a 'view from nowhere', and readers will be better able to interpret the review if they know why it was done and for which sponsor.

If a review is published, the process for reporting funding and conflicts of interest as set out by the publication concerned should be followed.

## Discussion

We have developed these publication standards for meta-narrative review (which we view as synonymous with meta-narrative synthesis) by drawing together a range of sources - namely existing published evidence, a Delphi panel and comment, discussion and feedback from a mailing list, training sessions and workshops. We hope these standards will lead to greater consistency and rigor of reporting and, thereby, make the outputs of meta-narrative reviews more accessible, usable and helpful to different stakeholders.

This publication standard is not a detailed guide of how to undertake a meta-narrative review. Other resources, both published (see Introduction) and in preparation, are better suited for this purpose. These standards have been developed as a guide to assist the quality of reporting of meta-narrative reviews and the work of publishers, editors and reviewers. As part of the RAMESES project, we will be developing and disseminating both training materials and quality standards for meta-narrative reviews [14].

Because meta-narrative review is used for a broad range of topics and questions, and because it involves making judgements and inferences rather then checking against or following a technical checklist, it is impossible to be prescriptive about what exactly must be done in a review. The guiding principle is that transparency is important, as this will help readers to decide for themselves if the arguments for the judgements made were reasonable, both for the chosen topic and from a methodological perspective. While we have encouraged review authors to provide detail on what they have done and how, we emphasize that these standards are intended to supplement rather than replace the exercise of judgement by editors, reviewers, readers and users of meta-narrative reviews. We have tried to indicate in each item where judgement needs to be exercised.

The sense-making focus of meta-narrative reviews means that detailed data may need to be reported in order to provide enough support for inferences and/or judgments made. While developing these publication standards, it became apparent that in some cases the word count limitations imposed by journals did not enable review teams to fully explain aspects of their review - such as how judgments were made or inferences arrived at. Alternative ways of providing the necessary detail may need to be found, such as online appendices or additional files available from authors on request.

Previous efforts to develop publication standards have sometimes been criticized for being too 'ivory-tower' and failing to take account of real-world problems faced by reviewers. In an effort to redress this problem in the RAMESES project, we sought from the outset to engage not just senior academics but also junior and mid-career researchers, practitioners, policymakers and publishers in the development of the standards and to capture real-life challenges of ongoing meta-narrative reviews as these emerged.

## Conclusions

We have developed these publication standards for meta-narrative review by drawing on a range of sources. Our hope is that these standards will lead to greater consistency and rigor of reporting and make the outputs of meta-narrative reviews more accessible, usable and helpful to different stakeholders. Meta-narrative review is a relatively new approach to evidence synthesis and with increasing use and methodological development, changes are likely to be needed to any publication standards. We hope to continue capturing and improving these publication standards, through our email list [15] and wider links and discussions with researchers and those who commission, sponsor, publish and use meta-narrative reviews.

## Abbreviations

RAMESES: Realist And MEta-narrative Evidence Syntheses: Evolving Standards

## Competing interests

The authors declare that they have no competing interests. The views and opinions expressed therein are those of the authors and do not necessarily reflect those of the HS&DR program, NIHR, NHS or the Department of Health.

## Authors' contributions

GWo carried out the literature review. JB searched the literature for meta-narrative reviews. GWo, TG, GWe and RP analyzed the findings from the review and produced the materials for the Delphi panel. They also analyzed the results of the Delphi panel. GWo, TG, GWe and RP conceived of the study, and participated in its design. GWo coordinated the study and ran the Delphi panel. All authors read and approved the final manuscript.

## Pre-publication history

The pre-publication history for this paper can be accessed here:

http://www.biomedcentral.com/1741-7015/11/20/prepub

## Supplementary Material

Additional file 1 **A Comparison between meta-triangulation and meta-narrative review**. This table compares the differences between meta-triangulation and meta-narrative review along nine dimensions. Click here for file
