# Supplementary material for: RAMESES publication standards: meta-narrative reviews
Source: BMC Med. 2013 Jan 29;11:20. doi: 10.1186/1741-7015-11-20 (PMC3558334; doi:10.1186/1741-7015-11-20)
Supplement: Additional file 1 — A Comparison between meta-triangulation and meta-narrative review. This table compares the differences between meta-triangulation and meta-narrative review along nine dimensions. [file 1741-7015-11-20-S1.doc]

### Additional File 1: A Comparison between meta-triangulation and meta-narrative review

| **A COMPARISON BETWEEN META-TRIANGULATION AND META-NARRATIVE REVIEW** | | |
| --- | --- | --- |
|  | **META-TRIANGULATION REVIEW [1]** | **META-NARRATIVE REVIEW [2]** |
| Purpose | To build theory. “Studying multifaceted phenomena characterized by expansive and contested research domains” | To build a rich, multifaceted picture of a complex topic, especially when a summary is needed for policy decisions |
| Philosophical basis | Constructivist (Kuhn’s philosophy of science) | Constructivist (Kuhn’s philosophy of science) |
| Intended audience | Academics | Policymakers |
| Type of insights | Analytic | Predominantly descriptive but recognises potential for analytic, theory-building insights |
| Examples of topics reviewed | Theoretical topics at high level of abstraction e.g. power, strategy | Policy and/or practice-relevant topics e.g. electronic records, knowledge translation |
| Empirical data | Included only as an aid to theorising | Included as substantive component of review |
| Unit of analysis | **Paradigm:** “the assumptions, practices and agreements among a scholarly community” | **Research tradition:** the historical unfolding of research on a particular theme by a group of scientists, which occurs within a paradigm |
| Key stages | **GROUNDWORK**  Define phenomenon of interest  Focus paradigmatic lenses  **SEARCH**  Collect data interpretable from multiple lenses  **MAPPING PARADIGMS**  Plan paradigm itinerary (ordered use of different paradigmatic lenses)  Code data  Write paradigm accounts  **THEORY BUILDING**  Explore metaconjectures  Attain meta-paradigm perspective  Reflect critically on the process | **GROUNDWORK**  Assemble multidisciplinary team  Outline research question  Agree outputs with funder  **SEARCH**  Browse literature to identify the different research traditions in this topic area  Search within each tradition to identify seminal conceptual and theoretical papers  Search systematically for empirical papers  **MAPPING RESEARCH TRADITIONS**  Describe paradigmatic basis for each tradition  Highlight the ‘storyline’ of each tradition (key issues and discoveries as they unfolded)  Appraise and summarise primary studies  **SUMMARY / SYNTHESIS**  Summarise each research tradition separately, highlighting similarities and differences  View discrepancies as higher-order data; explain as contestation between paradigms  **RECOMMENDATIONS**  Consider implications for sponsor / audience |
| Quality principles | **Reflexivity:** Theorist should be fully aware of own assumptions  **Systematic cross-paradigm synthesis techniques:** e.g. paradigm bridging (seeking commonalities), paradigm bracketing (highlighting differences), interplay (exploring tensions); meta-theorizing (exploring patterns that span conflicting understandings) | **Pragmatism:** What to include is not self evident; protocol must be developed in an emergent way and with attention to needs of funder / audience  **Pluralism:** Include multiple perspectives and ask what we can learn from each  **Historicity:** Trace research traditions over time  **Contestation:** Use “conflicting findings” in a positive way to generate new insights  **Peer review:** Present emerging findings periodically to a critical external audience |

Reference List

1. MW Lewis, AJ Grimes: **Meta-triangulation: Building theory from multiple paradigms.** *Academy of Management Review* 1999, **24:** 672-690.

2. T Greenhalgh, G Robert, F Macfarlane, P Bate, O Kyriakidou, R Peacock: **Storylines of research in diffusion of innovation: a meta-narrative approach to systematic review.** *Soc Sci Med* 2005, **61:** 417-430.
